# Terminal 18q deletions are stabilized by neotelomeres

**DOI:** 10.1186/s13039-015-0135-6

**Published:** 2015-05-13

**Authors:** Roberta Santos Guilherme, Karen E Hermetz, Patrícia Teixeira Varela, Ana Beatriz Alvarez Perez, Vera Ayres Meloni, M Katharine Rudd, Leslie Domenici Kulikowski, Maria Isabel Melaragno

**Affiliations:** Department of Morphology and Genetics, Universidade Federal de São Paulo, Rua Botucatu 740, CEP 04023-900, São Paulo, Brazil; Department of Human Genetics, Emory University School of Medicine, 615 Michael Street Northeast, GA 30322, Atlanta, USA; Department of Biophysics, Universidade Federal de São Paulo, Rua Três de Maio 100, CEP 04023-900, São Paulo, Brazil; Department of Pathology, Laboratório de Citogenômica, Universidade de São Paulo, Avenida Dr. Enéas Carvalho de Aguiar 255, CEP 05403-000, São Paulo, Brazil

**Keywords:** Terminal 18q deletion, Breakpoint sequencing, Telomere healing, Telomere capture, Neotelomere, Stabilization mechanism

## Abstract

**Background:**

All human chromosomes are capped by tandem repeat (TTAGGG)n sequences that protect them against end-to-end fusion and are essential to chromosomal replication and integrity. Therefore, after a chromosomal breakage, the deleted chromosomes must be stabilized by retaining the telomere or acquiring a new cap, by telomere healing or telomere capture. There are few reports with molecular approaches on the mechanisms involved in stabilization of 18q terminal deletions.

**Results:**

In this study we analyzed nine patients with 18q terminal deletion identified by G-banding and genomic array. FISH using PNA probe revealed telomeric signals in all deleted chromosomes tested. We fine-mapped breakpoints with customized arrays and sequenced six terminal deletion junctions. In all six deleted chromosomes sequenced, telomeric sequences were found directly attached to the breakpoints. Little or no microhomology was found at the breakpoints and none of the breaks sequenced were located in low copy repeat (LCR) regions, though repetitive elements were found around the breakpoints in five patients. One patient presented a more complex rearrangement with two deleted segments and an addition of 17 base pairs (bp).

**Conclusions:**

We found that all six deleted chromosomes sequenced were probably stabilized by the healing mechanism leading to a neotelomere formation.

**Electronic supplementary material:**

The online version of this article (doi:10.1186/s13039-015-0135-6) contains supplementary material, which is available to authorized users.

## Background

While interstitial deletions involve two breaks in the same chromosome arm, terminal deletions are caused by only one break leading to genomic loss. Terminal deletions of the long arm of chromosome 18 produce a highly variable phenotype. Among the most commonly reported features are growth deficiency, microcephaly, facial and limb abnormalities, genitourinary malformations, neurological abnormalities, hearing abnormalities and developmental delay with intellectual disability [1,2]. The phenotypic variability is related to the heterogeneity of the deletion size and gene content [3]. The deletions vary in size, but proximal breakpoints have been mainly described within bands 18q21.2 to 18q22.2, and does not correlate completely with the severity of clinical findings [3-5]. The region 18q22-q23 has been implicated as critical in development impairment but a deletion in this region leads only to a susceptibility to the clinical features, since not all patients with this region deleted have the same clinical findings [4-6].

All human chromosomes are capped with around 3-20 kb of tandem repeat (TTAGGG)n sequences and, immediately adjacent to this region, there is a segment of around 100-300 kb, the telomere-associated repeat (TAR) sequences [7]. These TAR sequences share homology with other chromosome ends [8,9]. Chromosome specific DNA sequences are located proximal to the TAR sequences [10]. Since telomeres are essential for chromosomal stabilization after breakage, the deleted chromosomes must retain the telomere or acquire a new cap [11,12]. Two main mechanisms were proposed to stabilize chromosome ends with terminal deletions: (1) telomere healing in which telomerase directly adds de novo telomeric repeats to unique nontelomeric DNA and (2) telomere capture in which the telomere is captured from another chromosome due to homology of sequence by a recombination-based mechanism, resulting in a derivative chromosome [11,13]. It is assumed that sequence homology of TAR regions between non-homologous chromosomes may influence telomere capture events [8]. Due to the development of molecular techniques, a variety of cryptic telomeric aberrations has been identified and showed that many apparently pure terminal deletions are in fact terminal deletions stabilized as derivative chromosomes by telomere capture [2,14,15]. The mechanism by which telomerase first recognizes a telomere or a broken chromosomal end is unknown [13]. Human genome subtelomeric regions are of particular interest in clinical cytogenetics, since they are the most gene-rich regions in the entire genome [13,16]. Therefore, characterization of telomeric regions is important for our understanding of the relationship between chromosome structure and function [9] and because chromosomal rearrangements involving telomeres result in a number of clinical conditions, including intellectual disability [17].

Thanks to recent advances in molecular genetic techniques, a better characterization of terminal deletions is possible [18,19]. The studies have demonstrated that several terminal deletions considered simple are not as simple as first thought and can present microduplication, inversion or addition of some base pairs, or they are in fact interstitial deletions. Sequencing of 18q terminal deletions may also help to understand the clinical variability presented by the patients. In this study we studied nine patients with 18q terminal deletions using G-banding, array techniques and FISH with telomeric probe. In six patients breakpoint sequencing was performed to determine the mechanisms involved in the stabilization of the deleted chromosome.

### Patients studied

Nine patients (7 females and 2 males) with 18q terminal deletion, detected by conventional karyotyping using G-banding and/or genomic array, were selected for the study.

## Results

**Figure 1:**
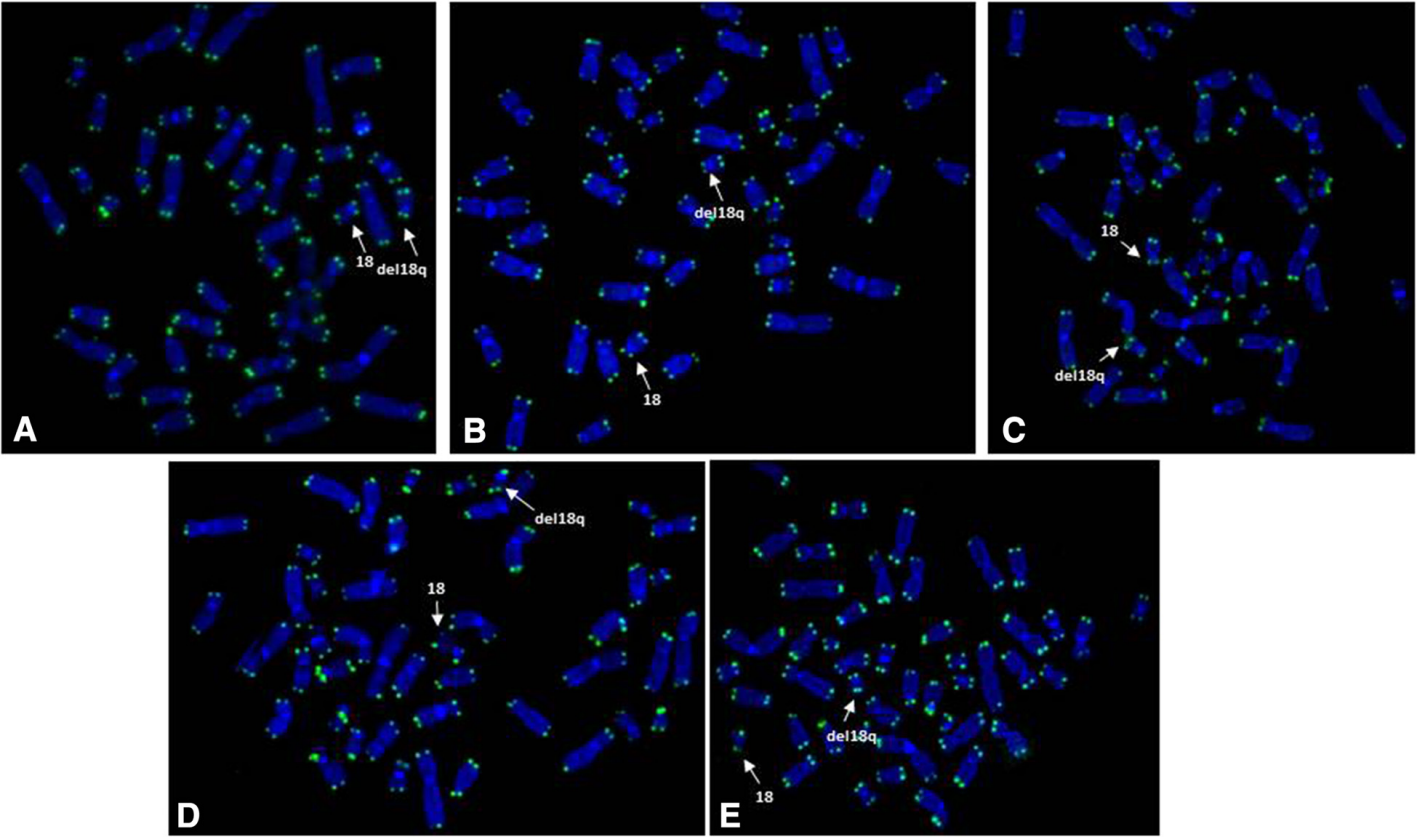
FISH results using Telomere PNA FISH Kit/FITC for patients with 18q terminal deletions showing telomeric signals in normal chromosomes 18 and in deleted chromosomes (arrows) in patients P1 **(A)**, P2 **(B)**, P5 **(C)**, P7 **(D)** and P8 **(E)**.

G-banding showed de novo terminal 18q deletions in all patients. FISH using PNA probes performed in seven patients revealed telomeric signals in both arms of the deleted chromosomes (Figure 1 and Table 1).

**Table 1:** **Breakpoint definition data using G-banding, SNP-array, custom array CGH and FISH results with PNA probe in patients with 18q terminal deletion**

**Patient**	**G-banding karyotype and SNP-array (Affymetrix)**	**Custom array CGH (Agilent)**	**Differences between the coordinates of breakpoints**	**FISH**
P1	46,XY,del(18)(q21.33)dn.arr 18q21.33q23(60,814,531-78,015,057) × 1	arr 18q21.33q23(60,814,632-78,015,147) × 1	101 bp	+
P2	46,XX,del(18)(q21.33)dn.arr 18q21.33q23(59,488,812-78,015,057) × 1	arr 18q21.33q23(59,488,412-78,015,147) × 1	400 bp	+
P3	46,XX,del(18)(q21.32)dn.arr 18q21.32q23(57,712,098-78,015,057) × 1	arr 18q21.32q23(57,714,859-78,015,147) × 1	2761 bp	+
P4	46,XX,del(18)(q21.32)dn.arr 18q21.32q23(57,818,459-78,015,057) × 1	arr 18q21.32q23(57,814,054-78,015,147) × 1	4405 bp	N
P5	46,XY,del(18)(q21.31)dn.arr 18q21.31q23(56,044,470-78,015,057) × 1	arr 18q21.31q23(56,046,905-78,015,147) × 1	2435 bp	+
P6	46,XX,del(18)(q22.1)dn.arr 18q22.1q23(62,772,720-78,015,057) × 1	arr 18q22.1q23(62,769,761-78,015,147) × 1	2959 bp	+
P7	46,XX,del(18)(q21.33)dn	N		+
P8	46,XX,del(18)(q21.32)dn.arr 18q21.32q23(58,641,269-78,015,057) × 1	N		+
P9	46,XX,del(18)(q21.32)dn.arr 18q21.32q23(58,938,942-78,015,057) × 1	arr 18q21.32q23(58,939,925-78,015,147) × 1	983 bp	N

(N) not performed, unavailable material; (+) signal telomere present by FISH.

**Figure 2:**
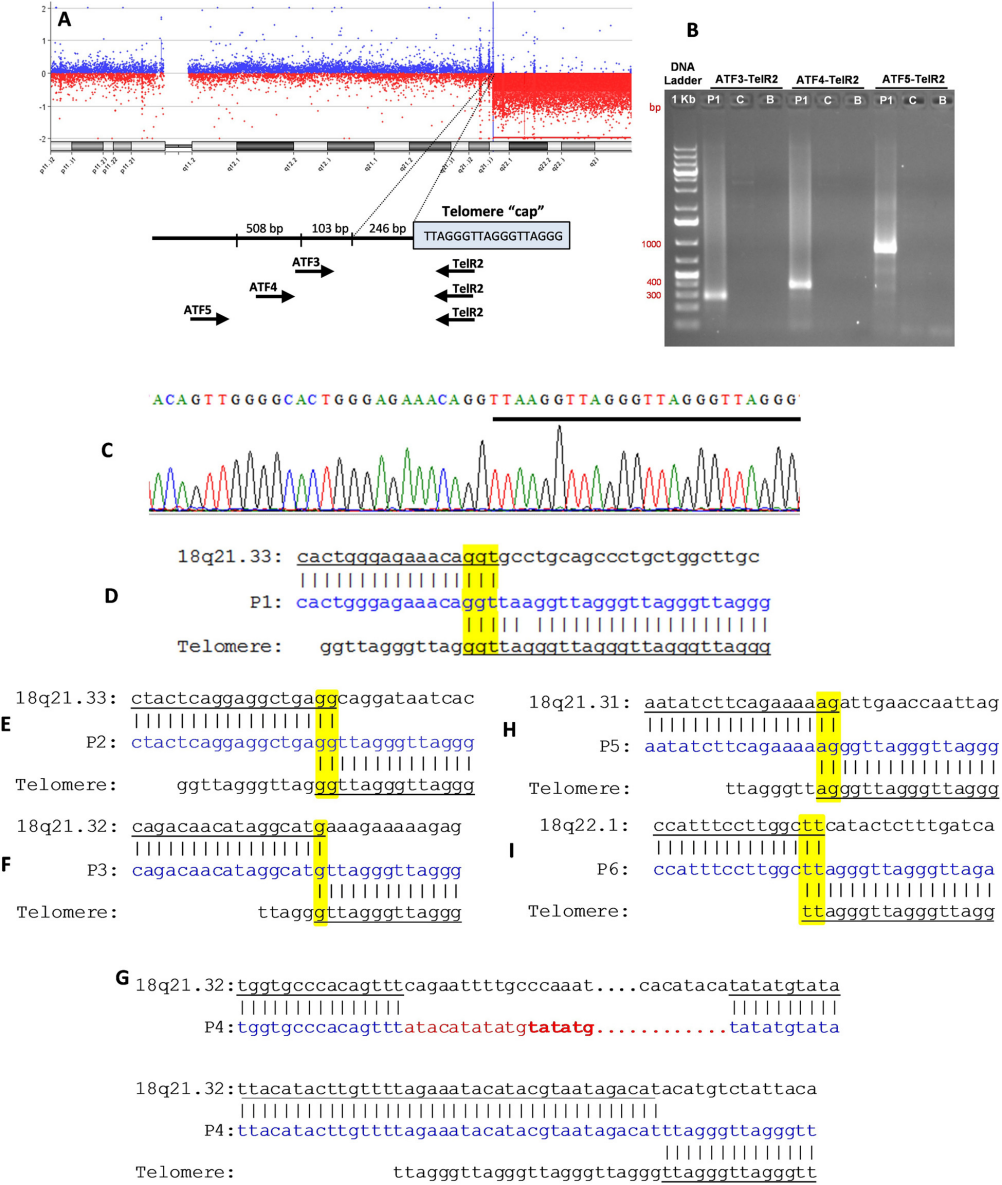
Steps in sequencing 18q terminal deletions for patient 1 **(A-D)** and data for the other patients **(E-I)**. **(A)** CGH-array profile in custom slide showing 18q21.33 terminal deletion for P1. Below, a schematic view of the breakpoint junction. The box, designated as “telomere cap” indicates the telomere (TTAGGG)n sequence. Black arrows show location of PCR primers in different combinations (ATF3, ATF4, ATF5 with TelR2); **(B)** PCR result in agarose gel 1.5% showing the fragment amplification using the three pairs of primers in P1 and no amplification in the male control (C) and blank (B); **(C)** Sequencing result showing the breakpoint in 18q21.33 and the beginning of telomere sequences (underlined); Alignment of the PCR products to the normal 18q sequence (above) and telomere sequences (below) showing microhomology (yellow) of 3 bp for P1 **(D)**; 2 bp for P2 **(E)**; 1 bp for P3 **(F)**; 2 bp for P5 **(H)** and 2 bp for P6 **(I)**; and a complex rearrangement with an interstitial deletion with addition of 17 bp (red) followed by normal 18q sequence before the telomeric sequences, without microhomology, for P4 **(G)**.

The data from SNP-array (for 8 patients) and from custom CGH-array (for 7 patients) are presented in Table 1. High resolution CGH-array narrowed the region around chromosome breakpoints to a few hundred base pair. For six patients (P1 to P6), breakpoint definition was performed and sequencing data from the PCR products were aligned to the reference genome. In these patients, telomeric sequences (TTAGGG)n were added directly after the 18q breakpoint (Figure 2). A 100 bp region each side of the breakpoints was chosen to verify the presence of repetitive elements around them. In P1, the sequencing junction revealed 3 bp (genomic coordinates: 60,814,620-3) microhomology at the breakpoint between the chromosome 18 reference sequence and the telomeric DNA sequence (Figure 2A-D), without repetitive elements around the breakpoint. Patients P2, P5 and P6 presented 2 bp of microhomology at the breakpoint (genomic coordinates: 59,487,156-8; 56,048,454-6 and 62,768,931-3, respectively) (Figures 2E, H and I). Around their breakpoints, repetitive elements SINE/*Alu* (for P2), LINE/L1 (for P5) and DNA/hAT-Tip100 (for P6) were found. In these patients the precise site of healing could not be precisely determined due to presence of two or three nucleotides of microhomology (yellow) that are shared between the genome reference and the telomere sequence in the breakpoint region. Patient 3 (P3) presents only 1 bp of microhomology (57,712,662), and repetitive elements were found around the breakpoint - DNA (hAT-Charlie) (Figure 2 F). In patient 4 (P4) the sequencing junction revealed three breaks, two of them resulting in an interstitial 8,625 bp deletion (57,815,639-57,824,623) with the addition of 17 nucleotides, not aligned to the 18q reference genome, followed by a normal sequence of chromosome 18q (57,824,264-57,824,310) with a terminal deletion where telomeric sequences were attached (Figure 2G). SINE/MIR sequences were found around the first breakpoint and LINE/L1 and simple repeats were found around the second breakpoint. There was insufficient material to perform array and sequencing for the other three cases (P7 to P9).

## Discussion

Recently, high-resolution genomic analysis in patients with 18q terminal deletions have revealed a high variability in breakpoints and deleted regions, pointing to a contiguous gene syndrome [16,20]. Array results showed breakpoints in four different bands in our patients: four out of eight at 18q21.32 (57,714,859-58,939,925), some very close to each other, two at 18q21.33 (59,488,412 and 60,814,632), one at 18q21.31 (56,046,905) and one at 18q22.1 (62,769,761).

In this study seven patients presented telomeric sequences (TTAGGG)n in the long arm of the deleted chromosomes as shown by FISH with telomere probe. The sequencing of 18q terminal deletions in six patients revealed that the breaks were probably stabilized by a healing mechanism in which the telomerase enzyme synthesized a *de novo* telomere in the truncated chromosome at the breakpoints creating a neotelomere. There are other cases described in the literature stabilized by the same mechanism in different chromosomes with terminal deletions [10,11,13,21,22]. Although the deletions stabilized by telomere capture from another chromosome forming a derivative chromosome reported in the literature presented with DNA single copy sequences from both chromosomes [10,15,23], the possibility of the capture of only the (TTAGGG)n telomere sequences of other chromosomes, simulating a neotelomere formation cannot be ruled out. Luo et al [24] studied three cases with 18q terminal deletion and observed that the breakpoints were distributed throughout the end of the chromosome, as previously described in other studies [20]. In one of the cases, the stabilization mechanism was consistent with NHEJ between double-strand breaks in two different chromosomes [24].

Many factors may contribute to make some regions of the chromatin more susceptible to breaks and rearrangements, such as the presence of enriched tandem repeats at chromosome ends, fragile DNA sequences, LCRs, concentration of particular repetitive sequences and motif sequences [24-26].

None of the six breaks sequenced in our patients were mediated by LCRs since they were absent in the breakpoint regions. LCRs were also not observed flanking the breakpoints in patients with 9q and 4p deletions [10,21]. The analysis of the region around the breakpoints revealed that only in our patient 1, repetitive elements were not found.

Ballif et al [27] characterized breakpoint regions in four patients with 1p36 terminal deletion and found that all the breakpoints fall within repetitive DNA sequence elements. Bonaglia et al [22] observed that only 13 out of 22 breakpoints in chromosomes with 22q13 deletion fell inside repetitive elements. Yatsenko et al [10] sequenced 43 breakpoints within the 9q34 region and the analysis of these junctions revealed that they were concentrated in regions with a high incidence of repetitive elements. Similarly, some of the breakpoints from our patients (P2, P4′s second break, P5 and P6) were located inside repetitive elements. However, it is unclear how repetitive elements participate in recombination events or DNA replication and repair [10].

Few (1 – 3 bp) or no microhomology was found in the breakpoints in our six patients who had their breakpoint sequenced. Similar lengths of microhomology were detected at breakpoint junctions sequenced from 14 terminal deletions of different chromosome ends [24]. In vitro studies have demonstrated that mammalian telomerase is capable of *de novo* telomere addition with as little as a single bp of homology or none to the template, suggesting that 3’ end pairing is not essential [28,29]. In fact, in a small proportion of terminal deletion breakpoints, microhomology was not found [12,13,21], similar to observed in our patient 4. Taking all these data, microhomology seems not to be essential for telomerase elongate truncated chromosomes.

The microhomology sequences in our five patients are homologous to (TTAGGG)n telomere repeats and may reflect the template-driven replication mechanism, in which the telomerase utilizes to replicate chromosome ends [30]. However, the site of healing cannot be precisely identified due to the microhomology between telomeric and breakpoint 18q sequences. Patient 4 presented an unexpected 18q terminal deletion with addition of 17 bp followed by an interstitial deletion in the first break and a terminal deletion. This event is probably the result of multiple steps: two breaks causing interstitial deletion with the addition of 17 bp that was stabilized by NHEJ (non-homologous end joining) followed by 18q normal sequence and a third break resulting in a terminal deletion stabilized by healing mechanism leading to the rearrangement observed. Similar to our study, other studies also found unexpected rearrangements at the breakpoint junctions such as interstitial deletion, additional nucleotides and inversion [10-12,22,24].

## Conclusion

Telomeric sequences must be present in both chromosome arm ends showing its importance to the stabilization of the chromosomes deleted. Breakpoint analysis is useful in elucidating the molecular mechanisms by which terminal deletions are stabilized. Few cases with terminal deletion 18q had their breakpoints determined at the base pair level. In our six patients with 18q breakpoint sequenced, telomeric sequences (TTAGGG)n attached directly in the breakpoint were found indicating chromosome stabilization by telomere healing mechanism originating a neotelomere. In four cases repetitive elements were present in the breakpoint junctions. One of the patients presented a more complex rearrangement suggesting that in rare cases stabilization of a terminal deletion is not as simple as first thought.

## Methods

### Classical and molecular cytogenetics

Chromosome analysis was performed first on 72-h lymphocyte cultures according to standard procedures. FISH (Fluorescence *in situ* Hybridization) using commercial probe Telomere PNA FISH Kit/FITC (Peptide nucleic acid) Dako® was performed according to manufacturing guidelines.

### Molecular studies

DNA was isolated from peripheral blood using Gentra Puregene kit (Qiagen Sciences Inc., Germantown, MD, USA). Samples were genotyped using the Affymetrix Genome-Wide Human SNP Nsp/Sty 6.0 array (Affymetrix Inc., Santa Clara, CA, USA) and data were analyzed with the Chromosome Analysis Suite (ChAS) Software (Affymetrix Inc., Santa Clara, Calif., USA), using annotation GRCh37/hg19. To define the breakpoints with a higher resolution, custom 8 × 60 K CGH-arrays (Agilent Technologies) were designed with probes targeting the breakpoints detected by clinical SNP-array (Affymetrix), using Agilents’ SureDesign program (https://earray.chem.agilent.com/suredesign/). The custom slides presented with a total of 62,976 probes targeted at 30 kb intervals surrounding the previously determined breakpoints. Control probes were also added for other chromosomes at a lower density. The unique identifiers (AMADIDs) for the array designs are 49352 and 67473 (designs available upon request). The experiments were performed according to manufacturing guidelines. The slides were scanned using DNA microarray Scanner with Surescan of high resolution (Agilent Technologies) and signal intensities were evaluated using Feature Extraction Standard Edition 6.5.0.58 (Agilent Technologies, Santa Clara, CA). Breakpoint’s analyses were performed using Agilent Cytogenomics software.

In order to amplify breakpoint junctions, unique sequence forward primers, complementary to the intact (non-deleted) 18q chromosomal region, were designed using Primer3 software (http://simgene.com/Primer3) (Additional file 1: Table S1). These primers were paired with a reverse primer complementary to the telomeric repeat sequence that has been used in other studies: TelR2 5’- TATGGATCCCTAACCCTGACCCTAACCC-‘3 (Flint et al, 1994; Luo et al. 2011). Long Range PCR (Takara®, Japan) was performed with 5.0 μl PCR buffer (10x), 4.0 μl dNTP (2.5 mM each), 5.0 μl of each primer (2 μM), 7.0 μl Betaine (5 M), 0.25 μl Ex Taq (5 U/μl) and 1.0 μl of DNA template (100 ng). PCR conditions for amplifying terminal deletions were: 94°C for 1 min; 10 cycles at 94°C for 30s, 65°C for 1 min (decreasing 1.0°C per cycle), 72°C for 3 min; 20 cycles at 94°C for 30s, 59°C for 1 min, 72°C for 3 min; and the final extension at 72°C for 5 min. PCR products were purified from 1.5% agarose gels using the QIAquick Gel extraction kit (Quiagen) and the breakpoint junctions were sequenced in ABI PRISM 3500xl genetic analyzer (Life Technologies). A male control DNA sample was used in parallel in every reaction and no amplification was obtained as expected. The sequence chromatograms were analyzed using BioEdit software.

DNA sequences from the PCR products were aligned to the human genome reference assembly (GRC37/hg19) using BLAT tool on the UCSC Genome browser (https://genome.ucsc.edu/). The regions flanking the breakpoints (200 bp) of each patient were analyzed using different programs to search for homologue sequence (BLAST), repetitive elements and low complexity sequences (RepeatMasker database version 4.0.5).

### Consent

Informed consents were obtained from the patients’ parents according to the Research Ethics Committee of UNIFESP (CEP 0389/11).

## Additional file

Additional file 1: Table S1. Primers used to perform the PCRs.
