# Supplementary material for: Terminal 18q deletions are stabilized by neotelomeres
Source: Mol Cytogenet. 2015 May 13;8:32. doi: 10.1186/s13039-015-0135-6 (PMC4427916; doi:10.1186/s13039-015-0135-6)
Supplement: Additional file 1: Table S1. — Primers used to perform the PCRs. [file 13039_2015_135_MOESM1_ESM.docx]

**Table 1-** Primers used to perform the PCRs.

| **Study** | **Primer name** | **Primers** |
| --- | --- | --- |
| Telomere | Tel-R2 | 5’- TATGGATCCCTAACCCTGACCCTAACCC -3’ |
| Patient 1 | ATC-F | 5’ - AACCTACCTGCCAAGTGTTTTACAAGAACCTA - 3’ |
| Patient 1 | ATC-F2 | 5’ - CACTAATTCCCCACTCCCTCAGATACATAAAG - 3’ |
| Patient 1 | AT-F1 | 5’- CCCAACAAGCCAAACACTTT - 3’ |
| Patient 1 | AT-F2 | 5’- TTTACAAGAACCTAGCTCTG - 3’ |
| Patient 1 | AT-F3 | 5’ - GGTCTGAGTGTCGACGCACCCTTTCTC - 3’ |
| Patient 1 | AT-F4 | 5’ - CCTGGCTCTGAAGCTCATCATGTTATC - 3’ |
| Patient 1 | AT-F5 | 5’ - GCATTGTGGTTATGGGACAACTGGAGAG -3’ |
| Patient 1 | AD1F | 5’- CCCAACAAGCCAAACACTTT-3’ |
| Patient 1 | AD2F | 5’- TCTCTCCCCTGCCTTCACTA -3’ |
| Patient 1 | AD3F | 5’- CCATTTGACCGTGGAAGTCT -3’ |
| Patient 1 | AD4F | 5’ - AAACACCCCGAGAAACTGTG -3’ |
| Patient 1 | AD5F | 5’ - GGGCAATGGAACCATACATC - 3’ |
| Patient 2 | ACC-F1 | 5’- GCCTCTCTGATTTATGCACTGATTTGTTCTAC -3’ |
| Patient 2 | ACC-F2 | 5’- TAGGACAGAATGATGAGATGTGAGAAGAGGTT -3’ |
| Patient 2 | AC-F1 | 5’ - GCAGGGTATTGCAATGAGGT - 3’ |
| Patient 2 | AC-F2 | 5’- GTAGCTGAAGTGAATAGTGATGATTAGGAC -3’ |
| Patient 2 | AG1F | 5’- GAGATTGCAGTGAGGCAACA -3’ |
| Patient 2 | AG2F | 5’- GCAGAGTGGGAAGACTGGAG -3’ |
| Patient 2 | AG3F | 5’- AAGCAGGGATGATGGAGTTG -3’ |
| Patient 2 | AG4F | 5’- AGTTCCCTGTCAAGCGTGTT -3’ |
| Patient 2 | AG5F | 5’ - GCAGGATCTTGCAGGGTATTGCAATGAG - 3’ |
| Patient 2 | AG6F | 5’ CTCTCAGAGAACCTGCAAGCACTCTCC - 3’ |
| Patient 2 | AG7F | 5’ - GTGTCCCTGGATGGAGCCATGATGACTC - 3’ |
| Patient 3 | TA4F | 5’ - GACACTGACAGTGGTTCCCAGGGAG - 3’ |
| Patient 3 | TA5F | 5’ - CCACAGGATGAAGGAGTGGGAATTTTGGC - 3’ |
| Patient 3 | TA6F | 5’- GGTGTGTCATTCCAAATAATCTAGGTACTC - 3’ |
| Patient 4 | V5F | 5’ - CTTTCTCCTGAGTTTCACATGGTTCGAGG - 3’ |
| Patient 4 | V6F | 5’ - GAACTGGAGAGTGCAGTCTCAAGGTCC - 3’ |
| Patient 4 | V7F | 5’ - CGTGTGAATTCAACTAAACTCTTCAAAGACC - 3’ |
| Patient 5 | RA-F1 | 5’ - CCTGCTGTCGGTAGTTGCGCT – 3’ |
| Patient 5 | RA-F2 | 5’ - CTCCCACGAGCTCTTGCTGAG – 3’ |
| Patient 5 | RA-F3 | 5’ - TGTGACGCTGCCCCGATTTCTC – 3’ |
| Patient 6 | TH-F1 | 5’ - GTCCCAAGTACTCAAGCCTCTCC – 3’ |
| Patient 6 | TH-F2 | 5’ - CACTCTTCCCTGCTCTGTGGGGTAC – 3’ |
| Patient 6 | TH-F3 | 5’ - CTCGCTCTGTTGCCCAACATGGAATG – 3’ |
| Patient 6 | TH-F4 | 5’ - CTCTCACAGACACCTGGCTGAGTCC – 3’ |
